# Understanding the perspective of traditional healers on their role within the Malawian healthcare system: a qualitative study in Thyolo District

**DOI:** 10.1186/s12906-026-05387-2

**Published:** 2026-04-18

**Authors:** Riya Master, Andrew Mguntha, Kimberly Baltzell, Gama Bandawe, Anneka Hooft

**Affiliations:** 1 School of Medicine, University of California, San Francisco, San Francisco, CA USA; 2 ENANDY Research Consultancy, Blantyre, Malawi; 3 School of Nursing, University of California, San Francisco, San Francisco, CA USA; 4 Malawi University of Science and Technology, Mikolongwe, Malawi; 5 Departments of Emergency Medicine and Pediatrics, University of California, San Francisco, San Francisco, CA USA; 6 Institute for Global Health Sciences, University of California, San Francisco, CA USA

**Keywords:** Traditional healers, Alternative medicine, Herbal medicine, Africa, Malawi, Rural medicine, Low resource, Infectious diseases

## Abstract

**Introduction:**

Malawi has a pluralistic health system, where people seek care from both formal (e.g., biomedical) and informal (e.g., traditional medicine) sources. An estimated 80% of the population utilizes traditional healers (TH) for care, particularly in rural areas. Given their strong community presence, TH may be able to bridge gaps in the healthcare system. However, data on barriers and facilitators to this relationship from the TH perspective are limited. Understanding TH perspectives on their role within the community is critical to integrating TH into the biomedical system.

**Methods:**

We conducted a qualitative study using semi-structured interviews of TH in Thyolo District, Malawi, in May 2024. A cross-sectional survey of community members in the area was used to inform participant recruitment for the interview study. Interview participants were identified and recruited purposively using these survey data and information gathered by the local study team through engagement with the Traditional Healer Association of Malawi. Interviews were conducted in Chichewa, translated and transcribed in English, and coded for themes.

**Results:**

We interviewed a total of 29 TH: 21 male and 8 female, ages 25 to 84 years. Time in practice ranged from 8 months to 60 years. Major themes identified included: (1) Relationship-building for diagnosis and treatment; (2) Trust and willingness to collaborate with biomedicine; (3) Compensation for gaps in the biomedical system; (4) Recognition of causes and risk factors for infection; and (5) Leadership role in the community.

**Conclusions:**

In Malawi, TH demonstrate a strong desire for recognition, training, and integration into the biomedical system. Understanding of disease processes beyond traditional vs. biomedical illnesses is variable, but most TH trust biomedicine and feel comfortable referring clients. Given their respected role in society, TH may provide a useful adjunct to biomedical care, however, additional context from healthcare providers and patients is needed.

**Supplementary Information:**

The online version contains supplementary material available at 10.1186/s12906-026-05387-2.

## Introduction

Malawi has a pluralistic health system, where patients seek care from both formal/biomedical (e.g., public or private hospitals, pharmacies) and informal (e.g., traditional healers, unregulated shops selling medications) sources. According to the World Health Organization (WHO), a traditional healer (TH) is defined as: “a person who is recognized by the community where he or she lives as someone competent to provide health care by using plant, animal, and mineral substances and other methods based on social, cultural, and religious practices [1].” It is estimated that over 80% of people in African countries utilize TH for their care [2]. 

There is no formalized educational or training pathway to becoming a TH in Malawi. TH may be taught the practice by a family member or another TH, while others claim to have been called to their profession, guided by a spirit or deity [3]. Some TH consider themselves experts in herbal medications (herbalists), others in spiritual problems and treatments (spiritualists), others as specialists in divination to determine the cause of a problem (diviners), some serve as informal midwives (traditional birth attendants), and many provide a combination of services or consider themselves general practitioners of traditional medicine [4]. This is in contrast to biomedicine (i.e., Western medicine, allopathic medicine), which is commonly understood to describe the provision of health care through the use of scientific principles, primarily from the fields of biology or chemistry [5].

In Malawi, as in many countries in sub-Saharan Africa (SSA), there is a severe shortage of biomedical health workers relative to the population, with less than 0.019 physicians and 0.283 nursing and midwifery personnel per 1,000 population [6, 7]. Due to the widespread use of traditional healers in Malawi, these informal providers may present a unique opportunity to bridge gaps in healthcare between rural communities and the biomedical sector. Successful examples of collaboration between traditional healers and biomedicine to improve health outcomes have been demonstrated by other studies in the Global South, including the incorporation of TH into the HIV care continuum [8–11]. However, numerous barriers to these collaborations include distrust, negative attitudes, differing ideologies, and a lack of recognition of traditional healers within the biomedical space [12–17]. Understanding these viewpoints and perspectives of traditional healers is a critical first step in establishing joint healthcare initiatives with biomedicine in Malawi.

Malawi and other countries in SSA are estimated to have high rates of insect and animal-transmitted infections and are at high risk for outbreaks of emerging and re-emerging diseases; however, a lack of diagnostic resources and health infrastructure makes early detection difficult [18]. Laboratory testing capacity and treatment of these infections are concentrated in urban, central hospitals; thus, patients presenting to informal providers in rural areas may face large delays in reaching a biomedical referral center, leading to losses of valuable time and data that could be used to initiate early interventions and curb potential outbreaks [18–20]. Detection and surveillance capacity for these diseases in Malawi is behind World Health Organization Standards for the region [21], and the prevalence of these infections is poorly described.

There are currently no established mechanisms for the inclusion or recognition of TH within the Malawian biomedical system, despite indications that TH are willing to collaborate [14]. Given their ease of access, integration, respected role within their communities, and high utilization rates among the population, particularly in rural areas and villages, informal providers may have untapped potential for integration into the public health system, including support for early detection of concerning infections [22–26]. Before TH can be integrated as sentinel providers, particularly for infectious threats, it is important to determine how TH view their role within the biomedical healthcare system.

The primary goals of this qualitative study were to characterize TH perceptions of their roles within the biomedical system and their communities, and to provide additional context on their experiences with infectious diseases in practice. It also serves as the first step toward a future pilot study in which a small number of TH will be trained to act as surveillance providers for potential infectious outbreaks, in partnership with a microbiology lab at a local university in Malawi.

## Methods

### Study design and setting

This was a qualitative interview study of traditional healers in Thyolo District, Malawi, conducted in May 2024. Thyolo District is a primarily rural, geographically diverse, agricultural district in Southern Malawi with a population estimate of almost 460,000. It borders Mozambique, is home to Malawi University of Science and Technology (MUST) and is less than 20 km from Malawi’s second largest city, Blantyre, making it an ideal pilot site for the future study to develop an enhanced surveillance system given the presence of traditional healers in this area, access to a large patient population, and proximity to the microbiology lab at MUST.

### Surveys conducted in public gathering spaces

We used anonymous, cross-sectional survey data collected by our study team in-person via a convenience sample of adults recruited from the general public in village gathering spaces (e.g., at trading centers, near health facilities) throughout Thyolo District, with the primary purpose of identifying TH sites in the area to use for recruitment in the interview portion of the study. People in these local areas were approached by a member of the study team fluent in Chichewa and asked if they would like to participate in a brief survey. They were then verbally consented to and asked a short series of anonymous, structured survey questions on-site. These surveys included questions about healthcare preferences and the specific locations of informal provider practices, primarily TH sites, in the region. We surveyed 135 adults across the Thyolo region, with over 100 of these participants providing at least one traditional healer practice site with which they were familiar.

### Recruitment of traditional healers for qualitative interviews

Traditional healers for informant interviews were then identified and approached using their business contact information from the aforementioned survey results, in conjunction with local research team knowledge and familiarity with the region and culture (AM), and engagement of the Traditional Healer Association of Malawi. Potential TH participants were initially approached by phone or at their place of work to invite them to participate, then enrolled, with written informed consent obtained in person before study participation. We used purposive, maximum variation sampling to include healers of all genders who identified different areas of specialization across various geographic areas throughout the region, to ensure a range of input and perspectives. All communications with traditional healers were performed in Chichewa by trained, local study staff.

Participant enrollment was continued until thematic saturation was reached. This was determined by consensus among the study team as the point at which transcripts no longer revealed any substantial new or relevant information, and when the generation of additional themes was deemed sufficient based on the content of completed interviews [27]. 

### Data collection

We conducted interviews with individual TH who diagnose and treat patients in Thyolo District and one initial group interview with key members of the Traditional Healer Association to obtain their input and feedback on the interview guide and topic before recruiting individual TH. Local members of the study team, trained in qualitative interviewing techniques and fluent in both English and Chichewa (the local language), used a semi-structured interview guide (Supplementary File 1) to conduct individual interviews in Chichewa, lasting approximately 1 h each. Interviews were conducted in a private area of the TH’s choosing at or near the location of their healer practice site. Interview questions aimed to understand TH perceptions of their roles in providing health services within their communities, particularly in relation to the diagnosis and management of infectious diseases. We also asked about their relationship with biomedicine. We included questions to gauge interest and identify TH who may be good candidates to serve as sentinel providers for the next phase of the study.

Interviews were audio-recorded and then transcribed and translated into English by bilingual (Chichewa/English) speaking research assistants. Transcription and translation quality were verified by AM and GB, both fluent in English and Chichewa.

### Analysis

Interview content was uploaded to Dedoose (Version 9.2.22, SocioCultural Research Consultants, LLC) and independently coded by two researchers (AH and RM) using thematic analysis to identify major themes. The goals of the larger study were used to organize and generate codes deductively, with additional codes generated inductively through an iterative approach. In cases of differing perspectives, discussion between the researchers and verification with interviewers continued until a consensus was reached. After initial analysis, the themes and subthemes and key codes used to generate them were reviewed further through discussion with the full study team, including two local team members familiar with cultural norms (AM and GB) to generate a conclusive list of themes and sub-themes was generated after discussion with the full team (AH, RM, KB, AM, and GB).

## Results

**Fig. 1:**
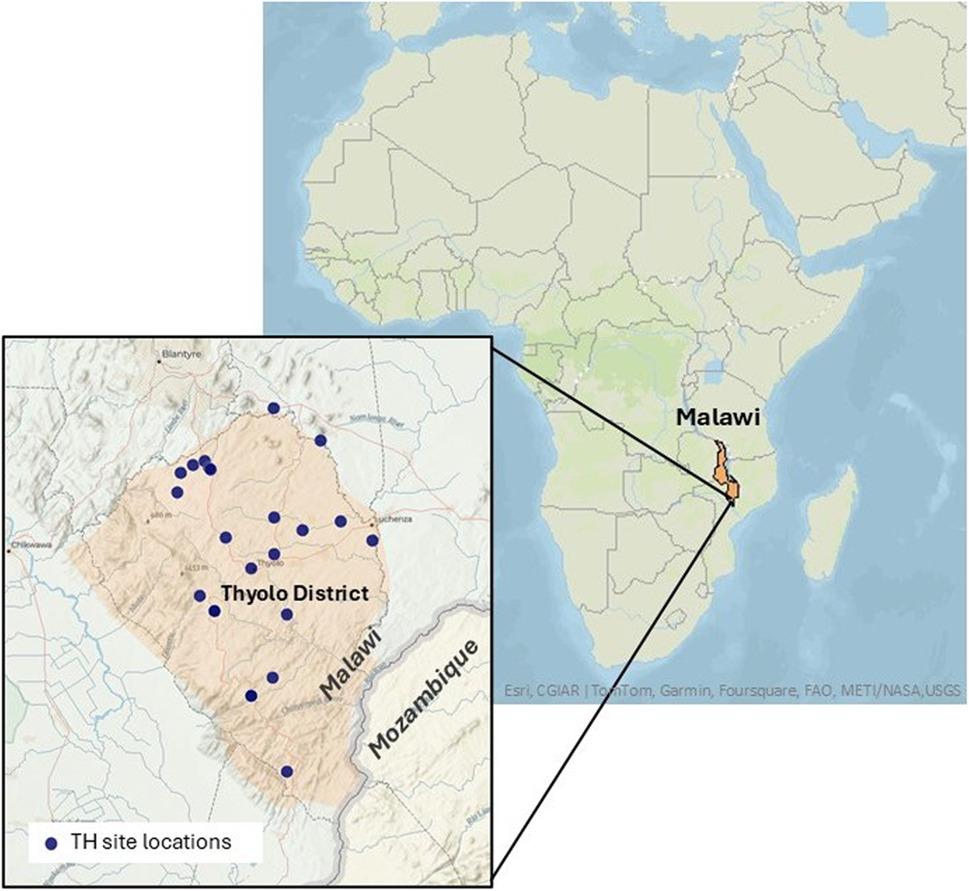
Map of traditional healer study sites within Thyolo District. Malawi (depicted in bright orange) is shown for context within Southeast Africa. Thyolo District is in the southeast of the country, bordering Mozambique. Locations of healer practices included in the study within the Thyolo District are depicted by navy blue dots

We interviewed a total of 29 traditional healers, 21 male and 8 female, 25 individuals, and 4 interviewed as part of the TH Association group. Age and time in practice ranged from 25 to 84 years and 8 months to 60 years, respectively, with practice sites located throughout Thyolo District (Fig. 1). Most participants identified as general traditional healers (*n* = 18), with training obtained from family members (Table 1). The following major themes were identified: (1) Relationship-building for diagnosis and treatment; (2) Trust and willingness to collaborate with biomedicine; (3) Compensation for gaps in the biomedical system; (4) Recognition of causes and risk factors for infection; and (5) Leadership and role in the community (Table 2).

**Table 1:** Traditional healer participant characteristics. Basic characteristics and practice specialty as identified by traditional healer participants

Characteristic	Key informants (*N* = 29)
Age (years), mean (SD)	60 (14)
Length of practice (years), mean (SD)	33 (15)
Gender, *n* (%):	
Male	21 (72)
Female	8 (28)
Training, *n* (%):	
Family members	17 (59)
Spiritual calling	10 (34)
Informal training	9 (31)
Some formal training	8 (28)
Healer specialty, *n* (%):	
General traditional healer	18 (62)
Herbalist	6 (21)
Spiritualist	9 (31)
Diviner	4 (14)
Traditional Birth Attendant	2 (7)

*SD *standard deviation

**Table 2:** Themes, subthemes, and illustrative quotations

Themes and subthemes	Quotation (Specialty, gender, age in years)
Relationship-building for diagnosis and treatment	
Reliance on history/exam	*Firstly*,* I do not have examination tools. As I have already said*,* I am not a fortune teller*,* so I depend on a patient’s history*,* on what he or she tells me. From there*,* I can decide how to treat the client. So*,* from the history*,* if it’s something I cannot manage*,* I refer the client to the hospital (Traditional Healer*,* M*,* 52)*
Continuity of care	…*we just instruct someone to mix and just take maybe half a tumbler … we tell them to take [it] for 7 days and come back again … if it does not change*,* we give them another traditional medication. (Spiritualist/Herbalist*,* F*,* 64)*
Trial and error	*So*,* I just try to help … just a trial*,* [I do] not even guarantee that the person will [always] get well … the spirits are the ones that do the final part.* *(TBA/Diviner*,* F*,* 60)*
Willingness to collaborate	
Variation in cooperation with biomedicine	*[Care of the patients I refer] depends on the heart of the doctor that they meet at the hospital. Some doctors are open to working with others*,* while others are just the ‘know-it-all’. So*,* I guess my worry over patients I refer sometimes [doesn’t] get recognized. (Spiritualist*,* F*,* 76)*
Decreased governmental support	*Yes*,* at first*,* they could give us referral cards*,* [so] that after referring them they would be treated fast*,* the patient was not neglected. But now we are no longer given those referral cards… they would even give us gloves because they know there are some conditions where*,* even as traditional healers*,* we need gloves… So*,* we know that they respect us. I don’t know about these days when our job of helping women to deliver has been stopped. I don’t know if they still respect traditional healers as before (TH/TBA*,* F*,* 59)*
Similar goals	. *The best way that I see biomedicine can be useful to a traditional doctor like me is when they begin to recognize and support us*,* maybe with gloves and other needs that can make our work safer and commendable. They can also help us with civic education/training in some health aspects. We are handling the same patients that they also handle every day. Patients choose both sides*,* so the best is just to help each other by working together in a safer environment (TH*,* M*,* 68)*
Bidirectional referral	*Some doctors do send some patients to traditional healers when they see that their treatment is not effective. What we all need is for all people to be healthy. [The] government needs healthy people to help in developing the country. (TH/Diviner*,* M*,* 80)*
Compensation for gaps in the biomedical healthcare system	
Overcrowding of health facilities	*The hospital days are usually crowded with people seeking different medical attention. So*,* I can say the accessibility is limited; those being assisted are also limited because of several problems*,* including limited drug supply and limited government cash flows (TH*,* M*,* 54)*
Transport delays	*It is the patient who then tells me their problems*,* like a lack of transportation. So*,* when I have the capacity and resources I do*,* I even carry them on my motorbike to the hospital. (TH/Spiritualist*,* 60)*
Concomitant use with biomedicine	*Even medical practitioners (doctors) come to us seeking medical help. Even though they are well educated*,* they find us here in the village looking for traditional medicine*,* so they know the difference.* *(TH*,* M*,* 59)*
Privacy and stigma	*Women come with problems like [being] infertile or barren. I give them herbs that make them become fertile … Men come with different problems*,* but some include paralysis and underperformance [during sex]. That’s common in men. (TH*,* M*,* 52)*
Recognition of causes and risk factors for infections	
Variable definitions of infection	*Infectious diseases attack gradually. You may wonder*,* you stay with it for more days before you realize you are sick*,* while these non-communicable infections attack the person instantly and with force… (TH*,* M*,* 59)*
	*Infections are diseases transmitted from one person to another. These include syphilis*,* chancroid*,* and other conditions like spinal bifida and swelling of the umbilical cord area (TH/Spiritualist*,* F*,* 70)*
Understanding of transmission	*Some [diseases] are from food*,* others from reckless behavior*,* like not washing hands*,* or*,* in short*,* poor sanitation. Not having refuse pits*,* untidy homes. But washing [our] hands with soap will make us safe from several diseases. You eat a guava fruit without washing it*,* you never know what was on it before plucking and eating it.” (TH/Spiritualist*,* M*,* 60)*
	*[One gets infected by measles through] bewitching each other and borrowing each other’s clothes*,* especially with those infected (Spiritualist*,* F*,* 50)*
Treatability	*I challenge that if I fail to treat an infection*,* then even at [the] hospital they cannot manage. If it’s not a normal infection*,* then I advise them to go to traditional healers instead of wasting their time at the hospital. Particularly those connected with witchcraft. (TH/diviner*,* M*,* 80)*
	*If the symptoms are new*,* then we know that the infection is unusual. If I know that I have never seen that type of infection based on the patient’s presenting symptoms*,* I just tell them*,* “I should not waste your time*,* hurry and go to the hospital and tell them the symptoms*,* so that they can see what to do.” (Spiritualist*,* M*,* 52)*
Leadership and role in the community	
Financial support/resources	*Most of the time*,* people come with different health problems. They may come while crying. I help them*,* but what they give me in return sometimes is not enough*,* meaning that most of my work is free just to help people … because I was born with a soft heart. There are healers who send back patients because they do not have enough money*,* but for me*,* I cannot do that. (TH*,* M*,* 59)*
Community leader	*I am a citizen of this village; I was born and raised in this village… and to add more*,* I am the chief of this village now (TH/TBA*,* F*,* 59)*
Social support	*There are some women who come with marital problems*,* some they want a job opportunity*,* or maybe they want a child …* *(Spiritualist*,* F*,* 76)*

*TBA *Traditional birth attendant

### Theme 1. Relationship-building with clients for diagnosis and treatment

Traditional healers rely on taking a good history and performing a physical exam to diagnose and treat their patients. They leverage their proximity to clients and community presence to enable follow-up visits and a trial-and-error approach to determine whether their initial assessment and treatment were accurate. Traditional healers believe biomedicine has better diagnostic capabilities, thanks to access to disease-specific testing and technology that TH do not have. Many TH, particularly herbalists, reported lacking diagnostic tools and sending patients to the hospital first for a diagnosis to inform their own management.

This TH discusses using the hospital to provide a diagnosis to help them determine which herbal treatment to use:

“*I give help according to what they are presenting with*,* the presenting symptoms*,* the disease history*,* and the diagnosis from the hospital if they have any; all this helps me to know which herbal medication to give. If I know the type of herbs for that disease*,* I give [the medication]*,* but if I do not know*,* I refer the patient to the hospital again.”* (Herbalist, M, age 49)

Another TH details the process of follow-up and continuity of care with their clients to assess therapy efficacy and guide further management. This service is often not available to patients using biomedical facilities for care, given the long travel distances and resources needed to get to a more centralized clinic or hospital for care:

…*when someone comes*,* I look at him*,* and I know something went wrong somewhere. I get some herbs*,* put them on a leaf … Get some that he can add to his porridge and give [them to] him… to boil and drink … When he takes those*,* you will see him coming the following day*,* [telling me] he is okay. *(TH/Spiritualist/Diviner, M, age 84)

The importance of hearing the patient’s full history is highlighted here. Traditional healers often reported having a strong relationship with their clients and spending time with them to get to the root of their concerns, whereas some biomedical sites have limited staffing.

*“Through asking questions to the client*,* that’s our main diagnosis tool*,* because when you don’t ask*,* then the patient may be delayed health aid…”* (TH, M, age 54)

### Theme 2. Trust and willingness to collaborate with biomedicine

All TH who participated in the study reported that they believed biomedicine is necessary and can be trusted, aside from some negative effects stemming from staffing and resource limitations and recent public fears surrounding COVID-19 and vaccination. Traditional healers indicated that they often refer clients to biomedical facilities and use them themselves for care when they are ill. For example, this TH stated,

*“I went to seek medical assistance when I had diarrhea and heavy vomiting. I knew that it would be difficult to just depend on traditional medicine*,* so I went to a government clinic…and they gave me drips*,* and I was fine.”* (Spiritualist, M, age 63)

They also provided examples of bidirectional referrals and receiving patients sent by biomedical providers for further care. All TH who participated in this study expressed a desire to further formalize this relationship, and many (*n* = 10) provided examples of previous programs in which their collaboration with biomedicine was stronger and supported by the Malawian Ministry of Health. They emphasize that current government policies and a lack of recognition have made it more difficult for them to earn the respect of biomedical providers.

One TH discussed her opinions on biomedicine and doubts in the community that arose during the COVID-19 pandemic:

“*My opinion on Western medicine is that it is necessary … People started having doubts on Western medicine because of coronavirus*,* believing that it was a satanic plot to wipe out communities. To me*,* I did not believe that … I saw that there is no reason to fear an injection [of the COVID-19 vaccine] and I just received it.*” (Spiritualist, F, age 64)

When unfamiliar with the disease, TH typically refers patients to the hospital. Many remember having a more formal referral process in the past, which has changed in recent years.

*“At first*,* we had referral papers that we would give patients*,* but it seems that initiative just died a natural death for some reason … Those referral papers were helpful in that when they got to the hospital*,* they should know that they had been referred from here. And when coming back*,* [the patients] would have come here first*,* but now all this has stopped. So*,* we just tell [the patient] to go to a health facility*,* and when they are there*,* it is up to them…”* (Spiritualist, F, age 76)

However, some TH do not feel respected by biomedical providers and even recommend that patients not tell the hospital they visited a traditional healer due to fear of being treated unfairly.

“*We do not understand why traditional healers and Western medicine service providers do not find a way to work together because both of us have our interest in the same subject*,* the patient. The hospitals need the patient*,* [and]* *so do we. [For] both of us, our agenda is the same: to heal. So why can’t we tolerate each other? … It was discovered that Western medicine practitioners look down on traditional healers because they went to formal colleges and attended training courses in Western medicine*.” (Traditional Healer, M, age 65)

Some TH also commented on the decrease in support from the Malawian Government, and their decision to ban the use of traditional birth attendants (TBAs) for deliveries and the requirement that all pregnant people receive services at a biomedical facility.

“*Imagine how it happened with Traditional Birth Attendants. They were completely stopped instead of finding a better way to improve their practice through training them [and] learning their ways. But it just stopped.*” (TH, M, age 52)

“*Sometimes the Ministry of Health could invite us [to seminars]*,* especially in the past*,* but now it does not happen. In the past*,* they would take us for training on how we can refer a patient*,* but also [during] that time we could communicate and receive a patient from the hospital*,* and we could give them a patient*,* too.*” (Spiritualist, F, age 76)

Despite this, there is a strong desire to collaborate with health centers and further the care of patients and the community. Traditional healers feel they share similar goals to biomedical providers and want the public to have access to high-quality healthcare.

“*I am telling you that*,* we want the government hospitals together with us*,* we should be working together … agreement on modalities of how we can work together instead of sidelining each other … there should be coordination*” (TH, M, age 68)

### Theme 3. Compensation for gaps in the biomedical system

Traditional healers provide care to community members who may have difficulty accessing biomedicine or are concerned about stigma and judgment. Participants provided examples of how TH often provide transportation or accessibility for patients who cannot present to the hospital or do not have the resources to get to a biomedical facility or pay for the treatment they may need. Additionally, TH cite being called upon only by the Ministry of Health during public health emergencies, such as the COVID-19 pandemic, but then feeling forgotten once the crisis has passed. Traditional healers recognize the lack of resources and biomedical providers as a gap they can fill in the community.

Here, TH note the assistance they provide with transportation and more accessible, flexible payment options for treatment, since many hospitals and medications are not easily available to community members due to distance and financial costs. Despite government hospitals being free, there are often drug shortages that require patients to visit private pharmacies, which are significantly more costly.

*“If they do not have money*,* they just use a bicycle to carry the patient*,* [pushing] the patient slowly until they reach there. Because they don’t have money. It’s terrible to narrate these sad events. This is why we do assist them with what we can before they reach the hospital to help preserve the patient’s life on the way.”* (TH/Spiritualist/Diviner, M, age 84)

*“It all depends on government hospitals because they are free. That’s why we want the government to recognize the traditional healers*,* so that people can access medicine from traditional healers*,* because herbs are cheaper than Western medicines. But financially*,* you can’t access Western medicine anywhere unless you visit a government facility. Western medicine is expensive in pharmacies and other outlets*,* and an ordinary villager cannot afford it.”* (Herbalist, M, age 64)

This TH describes how, during recent measles outbreaks, they were recruited to advise people on precautionary measures. They felt their value was acknowledged only when biomedical providers needed something from them, when “situations are tough,” rather than truly considering them as important colleagues and collaborators.

*“Most times us traditional healers*,* we are considered useless by those who provide Western medicine-based healthcare. We are only considered when things are critical. But when there is peace*,* we are useless people.”* (TH, M, age 59)

Some healers also describe providing care to clients who may face stigma for sensitive issues such as sexual performance or infertility. One healer describes how some clients prefer his services and feel more comfortable due to facing discrimination at biomedical facilities:

“*A poor person and their appearance alone*,* they are left to die … That is why some people do not trust the hospital when there is help [there]*,* but because of profiling*… *In the hospital*,* you have patients with different financial statuses. You see others [who] will bring rice*,* and they are eating*,* hence*,* the doctors assist [them] better*,* mostly*,* those who are well [to] do and not poor. I believe they receive an extra incentive from the patient guardians who are rich.*” (TH/Spiritualist, M, age 60)

### Theme 4. Recognition of causes and risk factors for infections

Traditional healers primarily categorize illness as either spiritual/traditional or biomedical, thereby defining what constitutes an infectious illness, and the level of understanding of infectious pathophysiology is highly variable. Mechanisms of infectious disease transmission, such as respiratory and fecal-oral contact, were commonly described, but traditional explanations were also frequently used. Specific knowledge of germs, such as bacteria or viruses, and how they differ, was much less well understood. There was also a high degree of variation in what is considered “treatable” based on attribution of cause to traditional vs. biomedical causes of illness.

This TH describes how they believe many infections are caused by bewitchment and can be treated with traditional medicine. While TH universally believe diseases caused by spiritual or traditional causes, such as bewitchment, cannot be treated by biomedicine, disease states that fall into this categorization vary by provider. This healer describes successfully treating infections caused by bewitchment:

*“All I can say is that I treat infections. I have treatment for different infections. For example*,* when I give some treatments*,* the patients purge*,* then they look like they are drunk*,* then all infections are gone! Then I command that every [bad thing] that is within the body of the man should go out*,* whatever it is in his body*,* the bad omen goes out through defecation … so I just suggest that he was bewitched.”* (TH/Diviner, M, age 52)

While this TH reports not knowing what causes infections:

*“These Infections I should not lie… us as herbalists*,* we don’t know where they come from*,* but a healthcare facility can know because they have examination equipment.”* (Spiritualist, M, age 52)

While another specifically mentions understanding that microorganisms cause tuberculosis (TB):

*“The coughs that are spreading all over*,* it is when I cough out sputum*,* or I have TB and cough there*,* then that microorganism that causes TB will go and enter someone*,* that person will also start coughing*.” (Spiritualist, F, age 64)

Many TH emphasized poor hygiene or risky behavior as key to the transmission of infectious diseases:

*“Some people are not using condoms … whether it is a woman or a man*,* they will sleep with many partners without a condom. They will go there*,* sleep with that one*,* take a disease and transfer it to another just like that … All sorts of infections are there*,* through the bad behavior*,* the promiscuous behavior.*” (TH, M, age 68)

While others use traditional explanations:

*“There has to be a difference because there are some illnesses*,* even if you wash your hands*,* you still get the infection. For example*,* traditionally*,* epilepsy*,* when you are at the back of the one having convulsions and they pass flatus*,* you can get the disease as well. But if you stand at the front*,* you cannot.”* (TH, M, age 78)

Perceptions of treatability also varied among TH, with some describing consistent referral of common infections like malaria, tuberculosis, cholera, HIV/AIDs, and COVID-19 to biomedical facilities for treatment. In contrast, others endorsed their own ability to treat some of these same illnesses. For example, one healer describes referring a patient to the hospital for suspected malaria for diagnostic testing with biomedicine as a standard procedure.

“*On malaria*,* we cannot give a person medicine because we do not have the treatment for malaria…he needs to go to the hospital to be told the severity of the malaria and the treatment he will get*,* either quinine or LA (lumefantrine-artemether). Myself*,* I cannot do that.”* (Spiritualist, F, age 64)

However, this TH, on the other hand, says there is a traditional medicine for malaria that can also be used as an alternative, after the patient has been diagnosed at a biomedical facility:

“…*we just tell them that this could be malaria*,* and you should go to a health facility to get quinine*,* but we do have our own traditional medicine that is bitter like quinine*,* we give the person*,* and the person gets better.”* (Spiritualist, F, age 76)

### Theme 5. Leadership role in the community

Traditional healers report feeling well-respected by their communities and often provide social services, such as financial support or transportation assistance, to their clients. They also note the influence they have on the community’s perception of biomedicine and help sensitize people to the importance of public health interventions, such as the COVID-19 vaccine. Some even serve as village chiefs or local government leaders and report attending hospital meetings to advocate for their community and culture. They report assisting their clients with a variety of social and familial issues, including marriage and financial struggles, in addition to providing medical care.

This TH discusses providing socio-economic support to his clients:

*“Some come with unhappy pale faces*,* as if there is a funeral*,* or a child is sick*,* so I give them medicine and tell them to bring the patient*,* I give them money to use*,* I am not a greedy doctor.”* (TH/Diviner, M, age 52)

One TH explained how he tries to help community members understand the lack of healthcare resources when they are frustrated that they cannot get the care they need:

*“But the main problem that people don’t know is that the challenges at government hospitals are not caused by the doctors but rather [by the] government. For me*,* as a group Chief*,* I have a role to sensitize people on this. For instance*,* [health center in the area]*,* doesn’t have enough health workers … when the patient is brought at night and the health worker is not on duty*,* the people will still demand that the person should work … [and] he is the only health worker at the institution*,* and he is supposed to perform all the duties during the day and night*,* which may be exhausting. But the community will be infuriated for feeling ignored. But our role is to explain to these communities the shortage of health workers.”* (TH, M, age 54)

Others provide social support and health education to clients, including those with chronic illnesses, and emphasize their need to take their medications and not purely rely on traditional medicine for disease states that have a known biomedical treatment.

*“We had a case of a client who had rashes. When he called and explained his symptoms*,* I asked him if he went to the hospital to get his blood tested. But it was found that he had stopped taking [his] ARVs (anti-retrovirals). We always encourage patients on ARVs to take their drugs because if they stop*,* the drugs cannot work effectively in their bodies.”* (TH, M, age 54)

## Discussion

Traditional healers in Malawi acknowledge their reliance on and willingness to collaborate with biomedicine while highlighting their ability to address some existing health system resource gaps, given their role as leaders and trusted members of their communities. TH’s understanding of infectious disease states is highly variable, and further training is needed to improve recognition of conditions more concerning for emerging or re-emerging pathogens, for which public health systems may benefit from early detection, and for conditions that require antimicrobials or other treatments that can only be obtained at a biomedical facility. Traditional healers indicate a willingness to learn and a desire for more training and recognition from the Ministry of Health, so they can improve their skills and play a larger role in Malawi’s healthcare system.

Our findings are consistent with prior studies in SSA demonstrating that TH are open to collaboration and often view their services as separate or adjunctive to biomedical healthcare. While some studies argue that the use of traditional medicine can be dangerous, harmful, or cause delays in care [12, 15–31]. Many others describe traditional healers working effectively within the biomedical system or providing support to those undergoing biomedical care. Healers may not only serve as informal providers, but can also add social support and encouragement for those reluctant or having difficulty with the biomedical healthcare system, particularly for persons living with HIV [11, 32–38]. 

There are notable social and political barriers to collaboration with the biomedical system, which has led to TH’s frustration with the government’s lack of effort to bridge the divide. This is apparent in the 2007 decision by the Malawian Ministry of Health to ban the use of traditional birth attendants. A unilateral reduction or ban on the use of traditional healers, including TBAs, for care may not improve outcomes if biomedical facility access and service quality are not expanded simultaneously, given the complex dynamics and multiple factors that influence healthcare delivery, particularly for marginalized populations [39, 40–42]. As with the use of community health workers or other lay providers within the public health system, requiring some degree of regulation, registration, baseline training, and skills assessments for TH may mitigate harms stemming from the use of corrupt, deceptive, or underqualified individuals for care.

A prior study in rural Uganda found that TH were successfully engaged in a program to recognize and refer clients with signs of Bubonic plague [32]. Based on this and our current study findings, the inclusion of TH as sentinel providers within the biomedical system is feasible but will require additional training and investment by biomedical systems in Malawi. As TH already alluded to being called upon during the COVID-19 and other public health crises, providing preparation and training to recognize and assist during other potential threats is imperative to maximize the efficacy of their role in these processes. As indicated by variable responses on diagnosing and treating potential infections, TH need training on risk factors for emerging or re-emerging infections and on assessing which conditions are more likely to be easily transmitted or to require early detection and treatment to prevent sequelae or outbreaks. This will require cooperation and more formalized systems within Malawi’s current healthcare infrastructure, which may be difficult to pursue without additional funding and resources. More studies are needed to pilot this process at a limited number of TH sites and to determine how to make it as streamlined and cost-effective as possible prior to widespread implementation.

Our study had several limitations. First, we only included the perspective of traditional healers and did not assess the views of biomedical providers or clients. Thus, it is likely that TH interpreted their potential role and service to their communities more positively. Second, this study was conducted in one region of Malawi where an organized Traditional Healer Association exists, and many participants were members of this group. This may have led to the recruitment of more highly trained healers or those with more respect for their integrity versus others who do not participate in this organization.

## Conclusion

Traditional healers in Malawi respect and value biomedicine and are open to collaboration. They view themselves as filling gaps in the public healthcare system caused by financial hardship and limited accessibility for rural populations, including during crises such as the COVID-19 pandemic. More training and education on infectious diseases and outbreak detection are needed before integrating healers into the biomedical system to serve as sentinel providers for emerging and re-emerging infectious threats, and additional studies are required to explore the views of biomedical providers and patients on this process.

Abbreviations: SSA: sub-Saharan Africa; TBA: traditional birth attendant; TH: traditional healer; WHO: World Health Organization.

## Supplementary Information

Supplementary Material 1: Traditional healer semi-structured interview guide.

## Data Availability

The data used and/or analyzed during the current study are available from the corresponding author on reasonable request.
