# Supplementary material for: Understanding the perspective of traditional healers on their role within the Malawian healthcare system: a qualitative study in Thyolo District
Source: BMC Complement Med Ther. 2026 Apr 18;26:203. doi: 10.1186/s12906-026-05387-2 (PMC13224511; doi:10.1186/s12906-026-05387-2)
Supplement: Supplementary file 1 — Supplementary Material 1: Traditional healer semi-structured interview guide. [file 12906_2026_5387_MOESM1_ESM.docx]

**Supplemental File 1: Traditional Healer Interview guide**

1. Please tell me a little about your yourself and your background – age, gender, how long you have been in practice, what is your area(s) of expertise, where you are from, your education.
2. Please tell me about your practice.

- Prompts: What types of patients do you see (e.g. children, adults, elderly, women)? For what types of problems do they come to you? In how many locations do you work?

1. Have you sought care personally in a biomedicine facility in the last 2 years? IF so, please tell me about your most recent visit to a biomedical facility (clinic or hospital).

- Prompts: What symptoms or problems stimulated the visit? What advice or treatments were you given?

1. Please describe how your practice differs from a biomedicine practice.

- Prompts: variation in the care provided, types of conditions treated, accessibility, cost.

4b. What is your opinion in biomedicine in general?

- Prompts: Discuss provider competence, trustworthiness, effectiveness. Explore accessibility of services.

1. Please tell me what you know about infections, or describe what the term “infection” means to you?

- Prompts: Please describe the difference between illness caused by infection and those that are not caused by infection?

*Say to participant: For the purpose of this interview, we will define infections as illnesses that are caused by germs in the body and are contagious (i.e. can be passed on to others). One example of this is malaria, which is caused by germs in the blood passed to us and on to others through mosquitoes.*

1. What are common symptoms of infections that you see? How do people acquire infections? What are some other common infections with which you are familiar? Give some examples of how different types of infections might be treated. Have you seen patients for infections? If yes, what kind of service did you provide.
2. What aspects of a client’s history, symptoms, or examination would concern you for a more unusual infection or illness?

- Prompt: What signs or symptoms would worry you that a client has a problem you are unfamiliar with or not comfortable managing? What do you do in these cases?

1. Have you ever referred clients with a possible infection to the clinic or hospital for testing or treatment?

- Prompt: If yes, please tell me about an experience you have had with referring a patient. What symptoms did the patient have? How did the referral work? How was the patient received? Did you feel your concern was respected by the biomedical facility (hospital or clinic)?

1. We are interested in detecting possible new or unusual infections when they first arise in members of the community. To do this, we are looking for providers, such as yourself, who may have the first interactions with these clients. We would then have these providers send out an alert to our team who would come and collect a blood and nasal swab sample from the client for testing. These would be sent to a special lab for testing which takes some time and will only give us possible causes of infection, so you or your client would not be provided the results. But the testing will help us find evidence of new or unusual infections and develop a system to detect illnesses before they spread, either locally or beyond this. Your clients would also receive a malaria test as part of the study and would be provided with these results at time of testing.

Would you be interested in working with us on this project?

If yes, do you have any other questions about what this would entail and what your role would be in the project?

Do we have permission to contact you in the future to discuss this further if it seems your practice would be a good fit?

1. Please feel free to ask any additional questions or comments about this interview or the project.
